# A child presented with bilateral congenital constriction ring in lower extremity: a case report

**DOI:** 10.4076/1757-1626-2-7772

**Published:** 2009-07-23

**Authors:** Richa Jaiman, Ajay N Gangopadhyay, Dinesh K Gupta, Punit Srivastava, Vijay D Upadhyaya, Shiv P Sharma, Vijayendra Kumar

**Affiliations:** 1 Department of Surgery, SN Medical CollegeAgra-282005, Uttar PradeshIndia; 2 Department of Pediatric Surgery IMSBHU, Varanasi-221005, Uttar PradeshIndia

## Abstract

**Introduction:**

The congenital constriction ring of lower extremity is very uncommon and rare condition. The actual incidence in general population is not known. In English literature, very few cases are reported time to time as congenital constriction band syndrome associated with musculoskeletal disorder like congenital talipes equino varus. The lesion can involve skin only or goes to deeper structure up to bone, which can lead to gangrene of foot or auto amputation.

**Case presentation:**

We are presenting a case of bilateral congenital constriction ring in lower limb who presented at age of 4 year without any other associated congenital anomaly, simply managed by Z-plasty, which improves quality of life after physiotherapy.

**Conclusion:**

Congenital constriction ring of lower limb is extremely rare condition in children. Early diagnosis and management is mandatory, either in single stage or by stage procedure, to prevent auto-amputation of limb and to improve quality of life on feet.

## Introduction

The incidence of this condition in newborn is 0.1% [1]. The condition has different terminology in literature like Streeter dysplasia, amniotic bands, annular defects and anomalous bands that encircle, either partially or completely a digit or an extremity [2]. The lesion can involve skin only or goes to deeper structure up to bone, which can leads to gangrene of foot or auto amputation [3]. Other musculoskeletal disorders that may be present are clubfoot, synacdactly or acrosynacdactly, hypoplastic nails, hypoplastic fingers, pseudoarthrosis of underlying bones, absence of bones, peripheral nerve defects, distal lymphedema, intrauterine amputations, cleft lip and cleft palate and umbilical hernia [4]. Exact etiology of this syndrome is not known [5] but it may be caused by prenatal environmental factors and it appears to be result of excessive contraction of the uterine muscles and hemorrhages from marginal blood sinus [6]. This case is reported because of its extreme rarity and gratifying result if diagnosed and managed early and secondly it usually presents at birth or soon after birth but our patient presented at an age of 4 years.

## Case presentation

**Figure 1.**
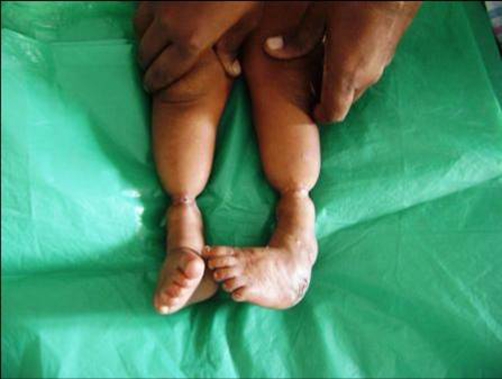
Bilateral constriction ring of lower extremity.

**Figure 2.**
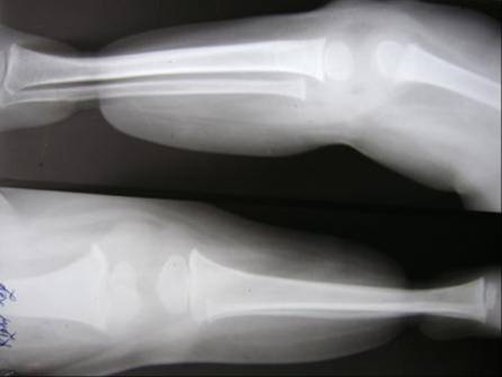
X-ray of both lower extremities showing severe soft tissue constriction with normal bone.

**Figure 3.**
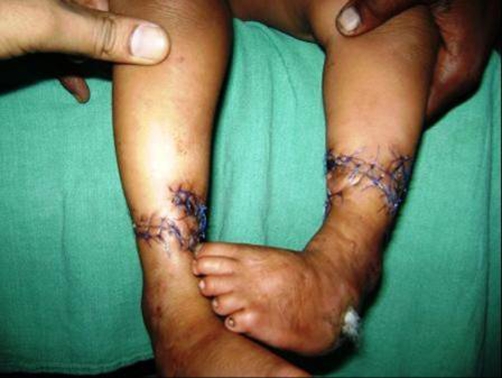
Postoperative photograph of both limbs after Z-plasty.

A 4-year-old Indian male child was admitted for treatment of constriction ring present in the both lower extremity about 3 cm proximal to ankle joint, in total circumference (Figure 1). There was discharging wound on the dorsum of left foot and rest soft tissue beyond the constriction ring was normal. Posterior tibial artery could not be felt at the constriction site in both limbs. Sensations were present in both limbs distal to constriction ring. All the toes in both limb was normal. There was no other associated abnormality in body. Roentgenogram of lower extremity revealed marked soft tissue constriction ring present in both limb without any bony abnormality (Figure 2). We managed our case by excising the constriction band and multiple Z-plasty. The postoperative condition was uneventful (Figure 3).

## Discussion

Amniotic band syndrome is a sporadic condition that may result in constriction bands, amputation and multiple craniofacial, visceral and body wall defects. It occurs in 1/1,200 to 1/15,000 live births. Most cases present with multiple congenital anomalies that are incompatible with life [7]. The incidence of congenital constriction bands of the extremities of childhood age in the general population is not known, but judging from the number of cases reported in the literature, the condition can be considered to be quite uncommon. Constriction bands are usually present since birth and may become more severe as the age of the child advances so it is necessary to manage them at the earliest to prevent complication. Usually the constriction bands are confined to the skin and the soft tissues, but sometimes they are deep enough to cut off the normal vascular and lymph return resulting in chronic edema of the limb [6]. Various degrees of the anomaly can occur, from a partial constriction ring to complete amputation of a limb [8]. The bands may be single or multiple, superficial or deep and can occur anywhere in the upper or lower extremity resulting in intrauterine gangrene and fetal amputations [8]. The various theories put forward about their origin are a primary constitutional defect of the germplasm, their production by amniotic bands or adhesions, a result of intrinsic circumferential necrosis of the superficial tissues, or due to some chromosomal disorder [6]. Patterson [2] in his study of 52 patients of congenital constriction rings had reported only two below knee amputations in addition to other musculoskeletal defects and classified them into four groups. Zych, et al [9] in 1983 reported a case of involvement of congenital bands, pseudoarthrosis & impending gangrene of leg, which was salvaged with multiple Z-plasty. Greene WB [1] in his study advised a one-stage release for circumferential congenital constriction bands which was performed in four extremities. Samra et al [3] in 2006 reported a case of severe constricting amniotic band with a threatened lower extremity in a neonate, which was salvaged with multiple Z-plasties after a 6-year functional follow up. The outcome of the disease depends on the gravity of the malformation. Termination of the pregnancy is usually proposed at the time of the diagnostic of severe craniofacial and visceral abnormalities, whereas minor limb defects can be repaired with postnatal surgery. In case of an isolated amniotic band with a constricted limb, in-utero lysis of the band can be considered to avoid a natural amputation [5].

Our case was operated in single stage, the one-stage release facilitated postoperative care, and there was no need for additional periods of anesthesia or for additional operations, which are necessary when this problem is treated with a release performed in two or three stages. Amniotic constriction band presents at birth and should be treated as early as possible, because the constriction bands are present a birth and become more pronounced as time passes. Recently constriction ring can successfully released by a minimally invasive endoscopic surgical technique avoiding severe limb dysfunction or foot amputation [7]. Congenital band may cause edema of the tissue distal to the band, they are distinguished from congenital lymph edema. If edema persists after surgical correction of the bands, excision of the edematous area may be necessary with direct closure or conversion of the overlying skin to thick, free (partial thickness skin grafts). Our case was reported because it presents at the age of 4 years even though there was no threatened complication like gangrenous changes, bony deformity or loss of sensation or function and it was treated in single stage by multiple Z-plasty. The patient was well in follow-up of three year periods when he was walking on own feet.
